# Preterm birth differentially impacts structural and functional connectivity of cortical gyri and sulci

**DOI:** 10.1016/j.dcn.2025.101647

**Published:** 2025-11-26

**Authors:** Elmehdi Hamouda, Wei Mao, Dan Xu, Keith Kendrick, Xi Jiang

**Affiliations:** a The Clinical Hospital of Chengdu Brain Science Institute, MOE Key Laboratory for NeuroInformation, School of Life Science and Technology, University of Electronic Science and Technology of China, Chengdu 611731, China; b China-Cuba Belt and Road Joint Laboratory on Neurotechnology and Brain-apparatus Communication, University of Electronic Science and Technology of China, Chengdu 611731, China

**Keywords:** Preterm birth, Neonates, Cortical folding, Gyri, Sulci, Synchronization

## Abstract

Preterm birth disrupts the gyrification process during the third trimester of pregnancy. Meanwhile, accumulating studies have highlighted the significant structural and functional differences between the folding patterns of cortical gyri and sulci, suggesting that they may play distinct roles in brain function. This study aimed to explore how preterm birth influences the structural and functional patterns of gyral and sulcal regions. Using a Developing Human Connectome Project (dHCP) open dataset including both full-term and preterm neonates (207 subjects), we parcellated each brain region into gyri and sulci based on the vertex curvature values. Structural connectivity was assessed via diffusion MRI (dMRI) images, and functional differences via fMRI BOLD signals using synchronization measures, nodal degree, and network-based statistics (NBS). Findings revealed that preterm birth reduces structural connectivity between gyri and lowers the ratio of intra-gyri/gyri-sulci connections. This ratio was significantly associated with gestational age, birth weight, and global synchronization. NBS analysis revealed a cluster of hypo-connections, mostly gyri-to-sulci connections. Overall, results suggest that preterm birth affects gyri and sulci differently, potentially disrupting their distinct functional roles, and offering new insights into prematurity’s impact on brain function.

## 1 Introduction

Premature birth is defined as birth occurring before 37 weeks of gestation or with a birth weight below 2500 g. It affects approximately 10 % of births worldwide, although the rate has been rising due to advancements in neonatal intensive care improving survival rate (Volpe, 2009, Blencowe et al., 2012, Chawanpaiboon et al., 2019). Individuals born preterm are particularly vulnerable to neurological, cognitive, and psychiatric impairments, with those born very preterm (<32 weeks, <1500 g) facing a 50 % risk of disability (D’Onofrio et al., 2013, Saigal and Doyle, 2008, Bulut et al., 2016, Hee Chung et al., 2020, Mwaniki et al., 2012). Additionally, preterm birth is strongly associated with various psychiatric disorders, including emotional disturbances, attention deficit/hyperactivity disorder (ADHD), and autism spectrum disorders (ASD) (Johnson and Marlow, 2011, Nosarti et al., 2012, Kuzniewicz et al., 2014, Franz et al., 2018).

The third trimester of gestation is a critical period for brain development, marked by a rapid increase in brain growth and structural maturation (Knickmeyer et al., 2008, Gilmore et al., 2012, Pfefferbaum et al., 1994). Preterm infants, who experience this crucial phase of neurodevelopment in an ex-utero environment, can be particularly vulnerable to hypoxia, ischemia, hemorrhage, and infections. These adverse conditions could trigger a combination of destructive processes and maturational disturbances, negatively impacting brain structure and function (Volpe, 2009, Saigal and Doyle, 2008, Mj, 2014). In particular, white matter abnormalities are among the most frequently reported consequences. Studies have documented alterations in Fractional Anisotropy (Rose et al., 2008, Li et al., 2015), Apparent Fiber Density (Kelly et al., 2020, Menegaux et al., 2020), and disruptions in the graph properties of the structural connectome (Sa de Almeida et al., 2021, Irzan et al., 2020). Functional brain connectivity is also affected, with studies highlighting alterations in resting-state functional connectivity networks (Cho et al., 2022, Eyre et al., 2021, Gozdas et al., 2018), and dynamic functional connectivity patterns (Ma et al., 2020, França et al., 2023), as well as a decrease in global mean synchronization (França et al., 2023). Some of these alterations might persist into adulthood and are linked to cognitive and emotional abnormalities (Menegaux et al., 2020, Papini et al., 2020).

Gyri and sulci are fundamental anatomical landmarks of the cerebral cortex. Traditionally, cortical folding has been thought to primarily serve as a mechanism to increase cortical surface area within the limited volume of the skull, thereby enhancing computational capacity (Zilles et al., 1988, Ronan and Fletcher, 2015). However, growing evidence suggests that gyri and sulci may play distinct roles in supporting cortical function, potentially acting as key building blocks of cortical morphology and function (Jiang et al., 2021, Deng et al., 2014, Uddin et al., 2010). Studies indicate significant differences in neuronal distribution between gyri and sulci regions (Hilgetag and Barbas, 2005). Additionally, axonal connectivity patterns reveal a structural preference: long-range axons are more densely concentrated within gyri compared to connections between gyri and sulci or between sulci themselves (Nie et al., 2012, Huang et al., 2009, Takahashi et al., 2012). Functional connectivity studies further support this distinction, showing strong connectivity among gyri, weak connectivity among sulci, and intermediate connectivity between gyri and sulci (Deng et al., 2014). The neural signal properties also differ between these regions. Gyri exhibit a narrow, low-frequency signal profile, whereas sulci display broader, high-frequency signals with greater complexity and temporal variability (Liu et al., 2019, Yang et al., 2019). Deep learning classifiers have been successfully used to differentiate between gyral and sulcal fMRI BOLD signals (Zhang et al., 2019, Ge et al., 2019, Jiang et al., 2020). These findings support the hypothesis that gyri function as central hubs for long-range information exchange between distant regions, whereas sulci may be more specialized for localized processing (Jiang et al., 2021, Deng et al., 2014, Zhang et al., 2020).

The gyrification process begins at the second half of gestation, and it is a crucial neurodevelopmental milestone of the third trimester (Zilles et al., 2013, Sun and Hevner, 2014). Consequently, preterm birth is particularly disruptive to this developmental process. Studies have reported altered gyrification in preterm-born individuals both in childhood and adulthood, with deviations associated with gestational age, cognitive function and mental health outcomes (Hedderich et al., 2019, Papini et al., 2020, Schmitt et al., 2022). If gyri and sulci serve distinct functional roles in cortical processing, preterm birth may disrupt the structural and functional differentiation between these regions. This altered differentiation could be a key factor underlying the cognitive and psychiatric problems associated with prematurity. It was therefore hypothesized that preterm birth would differentially influence structural and functional patterns of gyral and sulcal regions.

Therefore, this study aims to investigate the impact of preterm birth on the structural and functional patterns of gyri and sulci. We began by analyzing structural connectivity, hypothesizing that the denser structural links between gyri would be disrupted by preterm birth. We compared the average Structural Connectivity strength across gyri-to-gyri (GG), gyri-to-sulci (GS) and sulci-to-sulci (SS) connections. Then, to quantify the degree of gyral axonal density, we computed the ratio GG/GS and investigated the association of this ratio with the degree of prematurity. Inspired by cluster synchronization theoretical findings (Menara et al., 2020) that relate the intra-cluster/inter-cluster connectivity ratio to global synchronization, we examined the association between this ratio and mean synchronization, seeking to explain mechanistically the observed reduction in synchronization in preterm-born individuals (Franzça et al., 2024).

To further explore these synchronization reduction patterns at a coarser level, we first quantified synchronization levels within-clusters, i.e., separately within-gyri and within-sulci. Next, we employed Phase-Locking Value (PLV) to measure pairwise synchronization between brain regions, to identify where the reductions were most pronounced. We leveraged PLV matrices for further analysis because they focus on synchronization between two neural signals, without regard to their amplitude. First, we used Network-Based Statistics (NBS) to evaluate the alteration of functional connectivity in preterm birth and determine which specific connection types (GG, GS or SS) are significantly impacted. Second, we calculated nodal degree measures to assess whether gyral centrality in brain networks (Liu et al., 2017) was affected. This study is the first work to evaluate structural and functional patterns of cortical gyri and sulci in the neonatal brain, and how they are impacted by preterm birth.

## 2 Methods and materials

### 2.1 Schematic framework

**Fig. 1:**
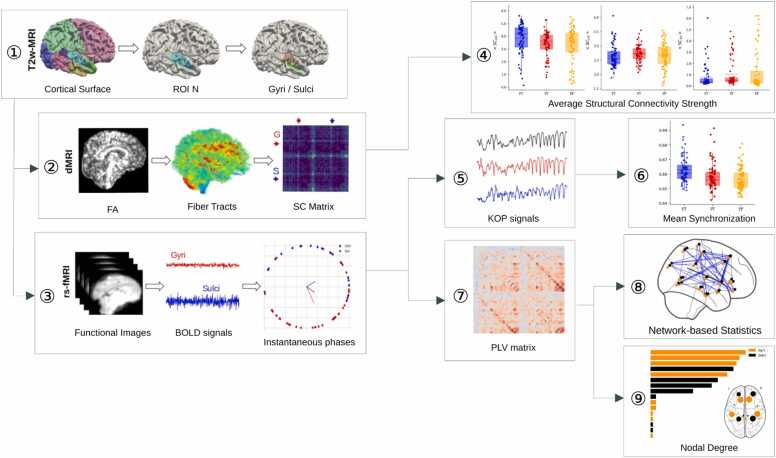
Schematic framework of the data analysis pipeline. 1- Parcellation of the brain with each region divided into gyral and sulcal parts. 2 - Calculation of the SC matrix from dMRI data. 3 - Extraction of instantaneous phases from BOLD fMRI signals to calculate KOP signals and PLV matrices. 4 – Calculation of the average structural connectivity strength. 5 – Calculation of the global, gyral and sulcal Kuramoto Order Parameter (KOP) signals from the instantaneous phases. 6 – Calculation of the Mean Synchronization (Global, Gyri, Sulci) as the mean of the KOP signals. 7 – Calculation of the Phase-Locked Value matrix from the instantaneous phases. 8 – Estimation of the altered functional connectivity using Network-based Statistics based on PLV matrices. 9 – Computation of the nodal degree from the PLV matrices.

The data analysis workflow is depicted in Fig. 1. The process started with the definition of regions of interest (ROIs) based on the dHCP neonatal basic atlas. Each region was further divided into gyral and sulcal components using the vertex curvature values (Fig. 1, panel 1). Subsequently, this parcellation scheme was employed to extract fiber tracts from diffusion MRI (dMRI) images, enabling the construction of Structural Connectivity (SC) matrices (Fig. 1, panel 2). From these matrices, the average SC strength was calculated for connections between gyri-gyral (GG), gyri-sulci (GS), and sulci-sulci (SS) pairs (Fig. 1, panel 4).

In a parallel stream of analysis, resting-state fMRI (rs-fMRI) data were utilized with the brain parcellation mask to obtain BOLD signals. The instantaneous phases of these signals were then extracted (Fig. 1, panel 3). The phase information, for each region at each time step, was leveraged for two key analyses: First, to compute time-varying Kuramoto Order Parameter (KOP) signals as a measure of the level of (global, gyri and sulci) synchronization (Fig. 1, panel 5), and second, to generate Phase-Locking Value (PLV) matrices representing pairwise synchrony (Fig. 1, panel 7). The mean levels of global, gyri and sulci synchronization were determined by averaging the KOP signals over time (Fig. 1, panel 6). PLV matrices were used to apply Network-Based Statistics (NBS) and to identify significant alterations in functional connectivity (Fig. 1, panel 8). Finally, the nodal degree for each brain regions was estimated from the PLV matrices, and an aggregated ranking of all regions was produced (Fig. 1, panel 9).

### 2.2 Participants

Participants were adopted from the second release of the Developing Human Connectome Project (dHCP) with authorization (http://www.developingconnectome.org/project/), which contain brain imaging data obtained from infants born and scanned between 24 and 45 weeks of age. The study was approved by the Research Ethics Committee reference number: 14/LO/1169 of the UK Health Research Authority and parental written consent for imaging procedures and data release was given.

Data from 437 neonatal participants with complete multi-modal T2-weighted structural MRI (T2w-MRI), diffusion MRI (dMRI), and resting-state functional MRI (rs-fMRI) were adopted. All participants were divided into three groups, based on their gestational age (GA) and post-menstrual age (PMA) at scan: 271 full term (FT) subjects (GA≥37 weeks and PMA≥37 weeks), 80 preterm subjects (PT) scanned at term equivalent age (GA≤37 weeks and PMA≥37 weeks) and 86 preterm subjects (PP) scanned “prematurely” (i.e., before reaching the term equivalent age) (GA≤37 weeks and PMA≤37 weeks).

Additionally, only the subjects with a radiology score RS≤4 were included. A radiology score of 5 indicates the presence of incidental findings that could influence the data analysis. Furthermore, due to the imbalance of the sample size of the full-term subjects compared to the preterm subjects, we used a Propensity Score Matching (PSN) method (Austin, 2011) to match the participants 1:1on the basis of scan age, sex and radiology score. PSN enabled us to get an equivalent sample size and reduce the effect of covariates, resulting in a sample of size N = 207, with 69 subjects for each group (FT, PT, PP).

Below is a summary of the sample statistics (Table 1 & Table 2). The FT and PT groups have the same scan age, while the PT and PP groups share the same birth weight but have slightly different gestational ages at birth.

**Table 1:** Distribution of Birth Age, Scan Age and Birth Weight in the three groups.

**Groups**	**Sex**	**Birth Age (weeks)**	**Scan Age (weeks)**	**Birth Weight (grams)**
FT	M	40.07 ± 0.88	40.97 ± 1.39	3.45 ± 0.4
	F	40.0 ± 0.99	41.38 ± 1.68	3.36 ± 0.35
PT	M	32.56 ± 3.83	40.43 ± 1.74	1.92 ± 0.87
	F	32.94 ± 3.59	41.44 ± 2.06	1.74 ± 0.63
PP	M	33.49 ± 2.59	35.43 ± 1.48	1.98 ± 0.65
	F	34.49 ± 2.88	35.98 ± 1.49	2.11 ± 0.66

**Table 2:** Differences in Birth Age, Scan Age, and Birth Weight across the three groups assessed using one-way ANOVA, with TukeyHSD test for post hoc analysis.

**Groups**	**‘FT = PT’**		**‘FT = PP’**		**‘PT = PP’**	
	**T stat**	**P Value**	**T stat**	**P Value**	**T stat**	**P Value**
Birth Age (weeks)	15.9	7.9e-33 (***)	16.64	1.3e-34 (***)	-2.019	0.045 (*)
Scan Age (weeks)	0.935	0.35	21.4	2.1e-45 (***)	17.66	5e-37 (***)
Birth Weight (grams)	14.9	2.3e-30 (***)	15.12	6e-31 (***)	-1.5	0.13

### 2.3 MRI data acquisition and preprocessing

T2w-MRI was acquired in sagittal and axial slice stacks with in-plane resolution 0.8 × 0.8 mm^2^ and 1.6 mm slices overlapped by 0.8 mm, TR/TE= 12000/156ms, SENSE factor 2.11 (axial) and 2.60 (sagittal). dMRI was acquired on 4 shells (b0: 20, b400: 64, b1000: 88, and b2600: 128) with SENSE factor 1.2, partial Fourier 0.86, resolution 1.5 × 1.5 mm, 3 mm slices with 1.5 mm overlap, and TR/TE= 3800/90ms. rs-fMRI was acquired with TE/TR= 38/392ms, 2300 volumes, and 2.15 mm isotropic resolution.

The preprocessing pipeline of T2w-MRI was described in (Makropoulos et al., 2018) including skull removal, tissue segmentation and surface reconstruction. The reconstructed triangular mesh cortical surfaces were mapped with curvature, brain region label and other features provided in the dHCP dataset. For dMRI, eddy current distortions correction and slight erosion of fractional anisotropy (FA) map via FSL were applied (Bastiani et al., 2019), The pre-processing of rs-fMRI is detailed in (Fitzgibbon et al., 2020). Both the cortical surface and pre-processed rs-fMRI volumes were registered to dMRI space via FSL-FLIRT.

### 2.4 Definition of regions of interest, structural connectivity and functional connectivity

#### 2.4.1 Regions of interests(ROIs)

We adopted the official brain atlas provided by dHCP for MRI data (Gousias et al., 2012) with 32 cortical regions. The brain atlas was registered to the T2w-MRI volume space and then mapped onto the cortical surface. We further adopted the vertex curvature value r to divide each cortical region into gyral part $r>r_{\mathrm{thr}}$ and sulcal $r<r_{\mathrm{thr}}$regions ($r_{\mathrm{thr}}=0.15$ was chosen as the threshold value as it yielded the best classification accuracy of gyral and sulcal signals (Mao et al., 2024)), resulting N = 64 ROIs in total. Note that previous studies (Jiang et al., 2021, Mao et al., 2024) have confirmed the effectiveness of such gyral and sulcal subdivisions within each brain ROI for investigating the differential role of gyri and sulci in cortical function. The list of ROIs is provided in Appendix Table 1.

#### 2.4.2 Structural connectivity (SC)

Deterministic fiber tracking via DSI Studio (Yeh et al., 2013) with tracking parameters (fine-tuned according to (Berman et al., 2009)) of 0.05 FA threshold, turning angle of 55°, fiber length restricted between 10 and 400, and fiber amount of 6 × 104. For each subject, the SC matrix (NxN) was calculated by using the ratio of the number of fiber tracts connecting each pair of ROIs over the total number of fiber tracts in the whole brain.

#### 2.4.3 Functional connectivity (FC)

The BOLD signals of rs-fMRI were mapped onto the cortical surface vertices by means of the nearest neighbor (or via trilinear interpolation from those of neighboring vertices if no nearest voxels are found).

### 2.5 Synchronization measures

#### 2.5.1 Kuramoto order parameter

The mean level of synchronization in the BOLD time series for each subject was quantified for the global, gyri and sulci. BOLD signals were filtered by a band-pass Butterworth second order filter in the range of 0.02–0.10 Hz. The robustness of our findings to the specific bandwidth across several narrower frequency ranges yielded consistent results. Hilbert transform was used to extract the instantaneous phases φ(t) from BOLD signals, which were considered after filtering as narrow-band signals described by $a\left(t\right)=A\left(t\right)\cos\left(\varphi \left(t\right)\right)$where A(t) is the instantaneous amplitude (or envelope), and φ(t) is the instantaneous phase. The first and last 20 s (50 TR) of the transformed BOLD signal was removed.

The level of synchronization was then computed using the Kuramoto Order Parameter (KOP), which measures the level of synchronicity of multiple oscillators and is defined as$\mathrm{KOP}\left(t\right)=\sum_{j=0}^{N}e^{i\varphi_{j}\left(t\right)}$where$\varphi_{j}\left(t\right)$ is the signal phase of an oscillator j (one region) at a given time (Kuramoto, 1984, Cabral et al., 2012).

#### 2.5.2 Phase-locked value (PLV)

To measure the phase synchrony between two regions, the Phase-Locked Value was used (Lachaux et al., 1999), which is a measure that quantifies the correlation of phase synchrony between signals based on their instantaneous phases φ(t). PLV is defined as:

$$\mathrm{PLV}=\frac{1}{N}\sum_{n=1}^{N}\exp\left(i\left[\varphi_{1}\left(n\right)-\varphi_{2}\left(n\right)\right]\right)\vee$$

where N was the length of time series.$\varphi_{1}\left(n\right)$and$\varphi_{2}\left(n\right)$are the instantaneous phase of two signals at time point n respectively.

### 2.6 Network-based statistics

Network-based Statistics (NBS) is a nonparametric statistical method to deal with the multiple comparison problem in conducting mass univariate significance testing among graphs. Compared with other statistical methods of a network, NBS could estimate family-wise error (FWER) correction for associations pairs between nodes, and is sensitive to detecting distributed networks with multiple connections. The FWER was controlled at the cluster level with p < 0.05. A total of 5000 permutations were performed, and the statistical threshold was set at T > 3.1 (Zalesky et al., 2010; Tan et al., 2022). We used the Brain Connectivity Toolbox (Rubinov et al., 2009) package (v0.6) for calculation, and Nilearn (v0.12) and pyCirclize (v1.10) packages for visualization in Python.

### 2.7 Functional hubs

The Nodal degree (Dnodal) of each node (brain region) was computed from PLV matrices. The Dnodal value is computed as the sum of the edge weights for edges that are incident to that node. The greater Dnodal of a node, the more important the node was in a network.

For each subject, the top 5 % nodes of which the Dnodal values were ranked were considered in the hub calculation. To examine the distribution of important nodes of FT, PT and PP subjects, the aggregated ranking percentage across all participants and determined the top 10 % of Dnodal nodes were evaluated as characteristic functional hubs of the group. Finally, the functional nodes with high ranking were projected into the topological brain map. Networkx (Hagberg et al., 2008) package (v3.3) in Python was used to compute the Dnodal values.

### 2.8 Statistical analysis

Statistical analyses were conducted using Welch’s ANOVA test to compare group means. This test was chosen because the assumption of homogeneity of variances was not met, as indicated by a significant Levene's test. Post hoc comparisons were performed using Tukey's Honestly Significant Difference (HSD) test to assess pairwise differences between group means. Mann-Whitney *U* test was used to compare categorical variables in (Fig. 5). A significance level of 0.05 was used for all tests. All analyses were conducted using the SciPy module (v1.14.0) in Python.

## 3 Results

### 3.1 Gyri-to-gyri structural connections are disrupted by preterm birth and are associated with clinical variables

**Fig. 2:**
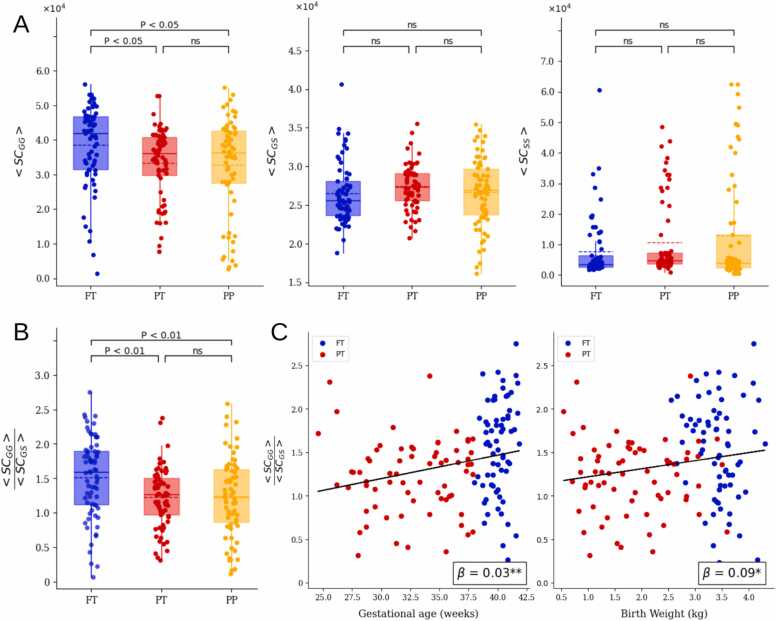
Comparison of gyri/sulci measures of average Structural Connectivity. (A) Average Structural Connectivity between gyri-gyri (GG), gyri-sulci (GS) and sulci-sulci regions. (B) Ratio of average gyri-gyri (GG) Structural Connectivity over the average of gyri-sulci (GS) Structural Connectivity. (C) Association between the ratio with gestational age and birth weight.

To examine the differential effects of preterm birth on gyri and sulci axonal connectivity strength, the average structural connectivity strength for gyri-to-gyri 〈SC〉, gyri-to-sulci $\langle {\mathrm{SC}}_{\mathrm{GS}}\rangle$, and sulci-to-sulci $\langle {\mathrm{SC}}_{\mathrm{SS}}\rangle$ connections across the full-term (FT), preterm at term-equivalent age (PT), and preterm scanned at birth (PP) groups were calculated (Fig. 2A). The analysis revealed that full-term neonates (FT) exhibited significantly greater average gyri-to-gyri (G-G) connectivity strength compared to both PT and PP groups, with no significant difference between PT and PP (Welch’s ANOVA: p = 0.011; Tukey HSD post-hoc: FT > PT (p = 0.0367), FT > PP (p = 0.0186), PT > PP (p = 0.0543)). In contrast, no significant differences were observed for gyri-to sulci (G-S) or sulci-to-sulci (S-S) connectivity strengths across the three groups (Welch’s ANOVA: G-S (p = 0.189), S-S (p = 0.0791)). These findings suggest that preterm birth selectively disrupts axonal connectivity between gyri, with no evidence of recovery by term-equivalent age.

Next, to quantify the degree of gyral axonal density relative to gyri-to-sulci, the ratio of average gyri-to-gyri (G-G) connectivity to gyri-to-sulci (G-S) connectivity across the three groups was calculated $\langle \mathrm{SC}\rangle /\langle {\mathrm{SC}}_{\mathrm{GS}}\rangle$(Fig. 2B) and a simple linear regression analysis performed to examine its relationship with prematurity-related variables (i.e., birth age and birth weight) (Fig. 2C). Results showed that the G-G/G-S ratio was significantly higher in the full-term (FT) group compared to both the preterm groups (PT & PP), with no significant difference between the PT and PP (Welch’s ANOVA: p = 0.003; Tukey HSD post-hoc: FT > PT (p = 0.004), FT > PP (p = 0.007), PT < PP (p = 0.065)). Furthermore, the ratio was positively associated with gestational age (slope=0.06, r = 0.32, p = 0.02) and birth weight (slope=0.06, r = 0.32, p = 0.02). These findings highlight a potential neurodevelopmental marker of preterm birth-related connectivity alterations.

### 3.2 Global synchronization is decreased by preterm birth and is associated with the ratio of intra-gyri to gyri-sulci connectivity

**Fig. 3:**
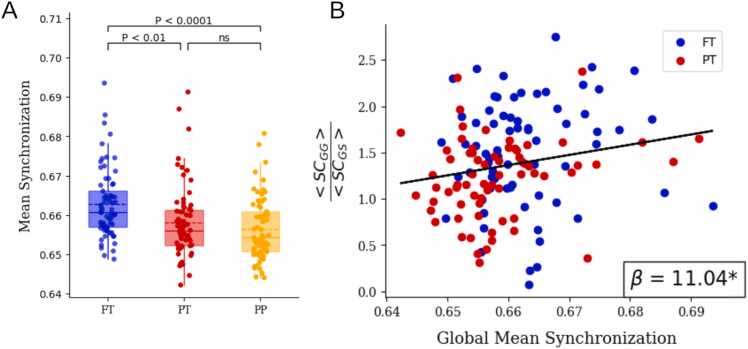
Global Mean synchronization. (A) Comparison of Global Mean Synchronization between FT, PT and PP groups (B) Association between Global Mean Synchronization and the ratio of average gyri-gyri (GG) Structural Connectivity over the average of gyri-sulci (GS) Structural Connectivity.

Previous studies have reported a reduced global mean synchronization in preterm neonates. We extend these results by including preterm infants scanned at birth (PP). More importantly, we look at the relationship between the ratio GG/GS and synchronization, motivated by the theoretical findings of cluster synchronization, where synchronization is being driven by a higher intra-cluster / inter-cluster ratio. Global mean synchronization was calculated using the Kuramoto Order Parameter, and found to be significantly higher in the full-term (FT) group compared to the preterm groups (PT & PP) with no difference between the latter (Welch’s ANOVA: p = 6e−5; Tukey HSD post-hoc: FT > PT (p = 0.004), FT > PP (p = 6e−5), PT > PP (p = 0.4625)) (Fig. 3A). Moreover, the relationship between the average G-G/G-S structural connectivity ratio and global synchronization was assessed and a significant positive correlation observed between the two variables (slope=3.25, r = 0.22, p = 0.0104) (Fig. 3B). These results suggest that the reduction in inter-gyri structural connection strength in preterm infants may contribute the observed attenuation in synchronization levels.

### 3.3 Preterm birth specifically affects sulci synchronization

**Fig. 4:**
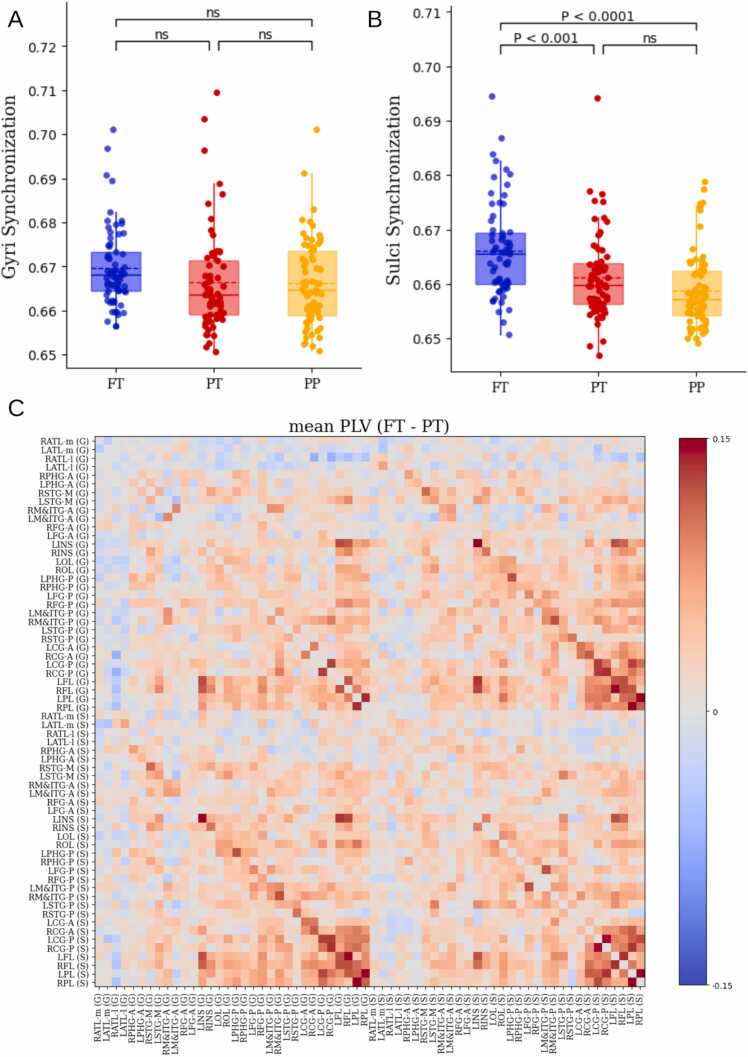
Gyri and Sulci Mean Synchronization. (A) Comparison Gyri Mean synchronization between FT, PT and PP groups. (B) Comparison Sulci Mean synchronization between FT, PT and PP groups. (C) mean Phase-Locked Value (PLV) matrix difference (FT-PT). Abbreviated notations for each region can be found in Appendix Table 1.

Next, to further investigate the synchronization reduction in preterm infants, we first assessed within-group synchronization in gyri and sulci, and which type of brain folding is more impacted. The mean synchronization levels of gyri and sulci were compared across the three groups to assess the differential impact of preterm birth on gyri and sulci synchronization. It was found that, for gyri synchronization, there was no significant difference between the three groups (Welch’s ANOVA: p = 0.0531) (Fig. 4A), while sulci synchronization was lower for the PT and PP groups (Welch’s ANOVA: p = 1e−6; Tukey HSD post-hoc: FT > PT (p = 0.0008), FT > PP (p = 3e−5), PT > PP (p = 0.1606)) (Fig. 4B). These results show that reduced synchronization was mostly within-sulci.

To explore the spatial patterns of synchronization at a coarser level and between different brain regions, Phase-Locked Value (PLV) was used to quantify pairwise synchronization between pairs of regions. PLV matrices were computed for each subject and the mean PLVs for each region pair was then compared between FT and PT subjects. The analysis revealed a significant decrease in phase synchrony in PT subjects compared to the FT group (red represent higher synchrony in FT neonates). The reduction was especially pronounced between sulci-sulci (SS) and gyri-sulci (GS), with the most significant decrease observed between the frontal and parietal regions (Fig. 4C). These findings suggest that reduced synchronization in preterm birth specifically affects sulci regions.

### 3.4 Preterm birth impacts functional connectivity between gyri and sulci regions

**Fig. 5:**
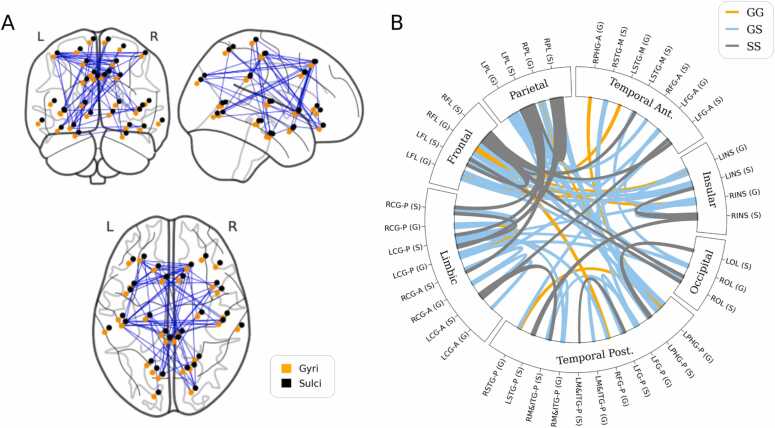
Difference of brain functional networks between FT and PT subjects the whole-brain network-based statistics (NBS). The identified sub-network (connected components) represents the difference of FCs between FT and PT subjects (FT > PT) on the PLVs (p < 0.05, FWE-corrected). (A) Regions of the sub-network and their topological maps projected into a cortical surface (B) Graph illustration of the sub-network. Abbreviated notations for each region can be found in Appendix Table 1.

To additionally examine the impact of preterm birth on pairwise synchronization as a measure of functional connectivity, and to specifically identify which type of connections were most impacted (GG, GS, SS), Network-Based Statistics method was used to identify hyper- and hypo-connected components in PT infants compared to FT infants. NBS has the advantage of detecting topological clusters, instead of single connections, reducing the bias toward spurious, isolated connections. PLV matrices were employed to detect sub-networks or topological clusters that showed significant differences between the groups. One significant cluster was detected in the FT > PT direction, with a statistical threshold of 3.1 (Tan et al., 2022). This cluster included both inter-hemispheric and intra-hemispheric connections, with the latter showing hemispheric asymmetry in the left hemisphere (Fig. 5A). The cluster contained a similar number of gyri and sulci regions, but most of the edges within the detected sub-network were G-S edges (in light blue), connecting gyri to sulci regions (Fig. 5B). These findings suggest that preterm birth specifically affects functional connectivity in gyri-to-sulci connections.

### 3.5 Preterm birth disrupts functional hubs

**Fig. 6:**
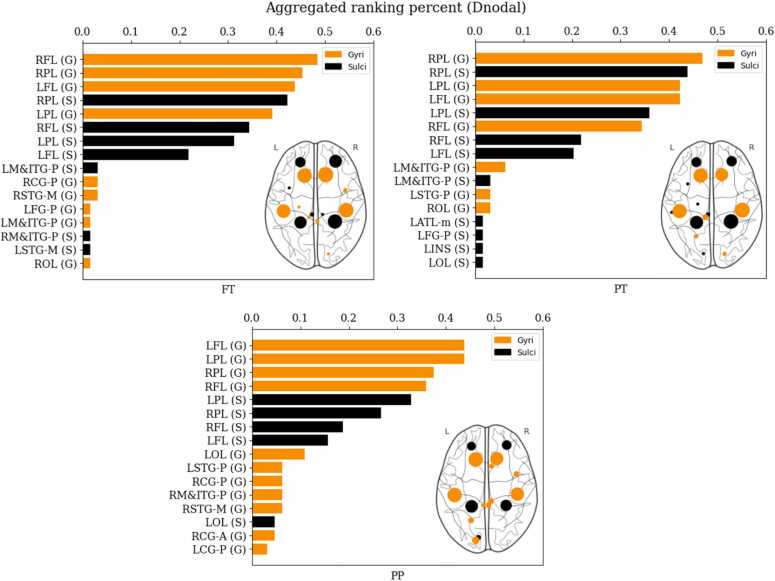
Functional Hubs with high nodal degree. Hubs based on the aggregated ranking percent of each node across 207 participants (69 each group) and their topological maps projected into a cortical surface obtained from the Dnodal value. Abbreviated notations for each region can be found in Appendix Table 1. The horizontal axes indicate percentage (%).

Finally, to assess the impact of preterm birth on network centrality of gyri regions in the functional connectome, the distribution of functional hubs across FT, PT, and PP subjects was computed. Using PLV matrices, brain regions were ranked according to their nodal degree value (Dnodal) in each subject. Gyri regions were found to have greater Dnodal values and ranked higher than sulci regions in all three groups, serving as more prominent functional hubs. However, some sulci regions ranked higher in PT infants compared to FT infants. Frontal gyral regions demonstrated the greatest decline in rank, whereas Parietal sulcal regions, displayed an upward rank shift. Conversely, gyri regions ranked higher than sulci in the PP group (Fig. 6). To compare the ranking of gyral and sulcal hubs, we calculated the number of such hubs within the top 5 % of nodal degree for each subject (e.g., a subject might have scores of gyri hubs: 2, sulci hubs: 1). A comparison of these counts between the FT and PT groups revealed no significant differences (Mann-Whitney *U* test: Gyri Hubs, FT > PT, p = 0.248; Sulci Hubs, FT > PT, p = 0.360), and contingency tables were used to report these findings (Table 3). These results indicate that while preterm birth impacts functional brain network hubs, it does not specifically affect gyri or sulci; rather, the influence is observed on specific regions.

**Table 3:** Comparison of participation of gyral and sulcal hubs among top 5 % hubs between FT and PT subjects.

**# of gyral hubs in the top 5 %**	0 (0 %)	1 (33 %)	2 (66 %)	3 (100 %)
FT	7	22	29	11
PT	8	24	29	8
**# of sulcal hubs in the top 5 %**	0 (0 %)	1 (33 %)	2 (66 %)	3 (100 %)
FT	17	26	21	5
PT	15	31	22	1

## 4 Discussion and conclusions

### 4.1 Summary and interpretation of results

This study investigated the impact of preterm birth on the structural and functional characteristics of cortical gyri and sulci patterns, and revealed several significant findings. The results indicate a differential impact of preterm birth on gyri and sulci. In preterm-born neonates, the structural connectivity strength between gyri was reduced. This relative reduction, quantified by intra-gyri over gyri-sulci ratio was associated with the degree of prematurity and decreased global synchronization. In contrast, the structural connectivity of sulci remained largely intact; however, both within-sulci synchronization and sulci-gyri pairwise synchronization were significantly affected. Additional functional connectivity analysis by network-based statistics confirmed a widespread reduction in connectivity, mostly affecting gyri-to-sulci connections.

Previous studies have reported distinct structural and functional patterns associated with cerebral folding (Jiang et al., 2021, Deng et al., 2014). The present study extends these findings to the neonatal brain, corroborating reports of denser white matter axons connecting gyri (Nie et al., 2012) and the centrality of gyri in functional networks (Liu et al., 2017). Our results demonstrate that this structural and functional differentiation emerges early in brain development. Furthermore, findings show that this differentiation is vulnerable to disruption by preterm birth, likely because prematurity interrupts key fetal developmental processes during the third trimester, such as gyrification and axonal formation (Zilles et al., 2013; Quinones et al., 2023). Abnormal gyrification is often reported in preterm-born infants and associated with cognitive difficulties (Papini et al., 2020, Schmitt et al., 2022). The results further support the functional model of differential role of gyri and sulci in brain function, and suggest that the pathologies associated with preterm birth may arise from a disruption in the dynamic interplay between gyral and sulcal structures which facilitates cortical processing. Within this framework, the observed reduction of integration and altered segregation of information in preterm-born individuals (Bouyssi-Kobar et al., 2019) can be interpreted as a disruption of the respective roles of gyri as information transmission hubs and sulci as local processing units.

A recent study (Franzça et al., 2024) using the same dataset demonstrated a reduced synchronization in preterm-born neonates, which was associated with higher Q-CHAT scores, indicative of more atypical social, sensory, and repetitive behaviors at 18 months of age. Our study identifies a potential structural marker that can explain this observation: a link between reduced synchronization and a global structural metric quantifying the relative density of gyral-to-gyral connections. This ratio was also associated with the severity of prematurity, evaluated by birth age and birth weight. This result aligns with the theoretical framework of cluster synchronization (Menara et al., 2020), which posits that a group of nodes with a high intra-cluster to inter-cluster connectivity ratio can drive global synchronization. A further condition for cluster synchronization, sufficiently distinct intrinsic frequencies, is also met in the cerebral architecture, as differences in the power frequency profile of BOLD signals have been reported between gyri and sulci (Liu et al., 2019).

Findings of reduced level of synchronization within-sulci but not within-gyri, despite the observed reductions in gyri structural connectivity but not sulci, can be plausibly explained through computational models on synchronization (Cabral et al., 2011). Studies have demonstrated that increasing global coupling of a computational model constrained by the brain’s structural connectome strengthens global synchronization, resulting in the gradual synchronization of all nodes, with the structurally less-connected nodes synchronizing last. This model explains our findings: under conditions of lower global synchronization, the less structurally connected sulcal regions are disproportionately impacted and exhibit lower levels of synchronization within the broader network. This also explains subsequent results: the pronounced reduction in PLV pairwise synchronization in gyri-to-sulci and sulci-to-sulci quadrants, and the disproportionate reduction in functional connectivity for gyri-to-sulci connections, as demonstrated by NBS.

Studies on preterm infants and adult consistently reported alterations in structural (Quinones et al., 2023; Sa de Almeida et al., 2021) and functional connectivity (Cho et al., 2022, Eyre et al., 2021). Our results build upon these findings by investigating these alterations from a new perspective, how they manifest differently across distinct cortical structures defined by folding: results indicate that gyri are predominantly affected in terms of structural connectivity, whereas sulci are more impacted functionally. Furthermore, the observed decreases and functional connectivity were most pronounced in frontal and parietal regions. This regional specificity aligns with prior studies, which have reported not only weaker white matter axons connecting frontal and parietal areas, but also a weaker connectivity in functional connectivity in frontal areas (Disselhoff et al., 2025) and between/within frontoparietal networks (Wehrle et al., 2018) These networks and regions are critical for motor, cognitive, and executive functions (Wheelock et al., 2018), which are often disrupted in preterm-born individuals (Allotey et al., 2018).

### 4.2 Implications and hypotheses

These findings provide further support for the hypothesis that gyri and sulci play differential roles in cortical function. Specifically, it is proposed that gyri function as hubs for information exchange, while sulci are more involved in local processing. The hypothesis that gyri are organized as a highly interconnected cluster that drives global synchronization in the system was explored. Preterm birth may disrupt this dynamic, leading to deviations in structural and functional connectivity. These disruptions could underlie the cognitive and psychiatric abnormalities observed in preterm-born individuals. Furthermore, the ratio of intra-gyri over gyri-sulci connectivity strength was associated with prematurity and reduced synchronization. If further validated, it can constitute a potential biomarker for early identification of neurodevelopmental risk within preterm-born populations. This ratio can also be mechanistically linked to synchronization, within the dynamical framework of cluster synchronization, making a mechanistic link between brain structure and function.

### 4.3 Limitations and future directions

Despite these important findings, there are several limitations to consider. The measures used in this study are global, and the observed differences could result from alterations specific to a few regions, rather than the hypothesized differential roles of gyri and sulci. Additionally, the parcellation used in the study was minimal and lacked detailed divisions within the frontal, parietal, and occipital lobes. However, the scope of the analysis focused primarily on the comparison of gyri and sulci, rather than differences in the cortex. Furthermore, it is possible that the brain of preterm infants may recover to normal structure and function with age, warranting similar analyses in adults born preterm. Finally, exploring potential associations with cognitive and psychiatric outcomes will be crucial to determine whether abnormal gyri-sulci differentiation contributes to intellectual deficits and psychiatric vulnerabilities (Jiang et al., 2023, Sui et al., 2023).

## 5 Conclusions

In summary, the current findings offer new evidence for the differential role of gyri and sulci in cortical function, investigating these gyri-sulci differences from a dynamical framework, where gyri act as a cluster that drives global synchronization. Furthermore, the study investigated the alteration of structural and functional connectivity in preterm-born individuals from a novel perspective, where preterm birth differentially impacts cortical regions, depending on their folding pattern. This suggests that the disruption of the distinct roles of gyri and sulci during brain development and function, can alter their dynamical interplay underpinning cortical function and potentially underlying brain disorders.

## CRediT authorship contribution statement

**Keith Kendrick:** Writing – review & editing, Supervision, Funding acquisition. **Dan Xu:** Methodology. **Wei Mao:** Methodology, Data curation. **Elmehdi Hamouda:** Writing – review & editing, Writing – original draft, Visualization, Methodology, Investigation, Formal analysis, Data curation, Conceptualization. **jiang xi:** Writing – review & editing, Supervision, Resources, Project administration, Funding acquisition.

## Declaration of Competing Interest

The authors declare that they have no known competing financial interests or personal relationships that could have appeared to influence the work reported in this paper.

## Figures and Tables

**Table:** 

Gyri Regions			Sulci Regions		
Index	Name	Abrv	Index	Name	Abrv
0	Right Anterior temporal lobe medial	RATL-m (G)	32	Right Anterior temporal lobe medial	RATL-m (S)
1	Left Anterior temporal lobe medial	LATL-m (G)	33	Left Anterior temporal lobe medial	LATL-m (S)
2	Right Anterior temporal lobe lateral	RATL-l (G)	34	Right Anterior temporal lobe lateral	RATL-l (S)
3	Left Anterior temporal lobe lateral	LATL-l (G)	35	Left Anterior temporal lobe lateral	LATL-l (S)
4	Right Gyri parahippocampalis et ambiens anterior	RPHG-A (G)	36	Right Gyri parahippocampalis et ambiens anterior	RPHG-A (S)
5	Left Gyri parahippocampalis et ambiens anterior	LPHG-A (G)	37	Left Gyri parahippocampalis et ambiens anterior	LPHG-A (S)
6	Right Superior temporal gyrus middle	RSTG-M (G)	38	Right Superior temporal gyrus middle	RSTG-M (S)
7	Left Superior temporal gyrus middle	LSTG-M (G)	39	Left Superior temporal gyrus middle	LSTG-M (S)
8	Right Medial and inferior temporal gyri anterior	RM&ITG-A (G)	40	Right Medial and inferior temporal gyri anterior	RM&ITG-A (S)
9	Left Medial and inferior temporal gyri anterior	LM&ITG-A (G)	41	Left Medial and inferior temporal gyri anterior	LM&ITG-A (S)
10	Right Lateral occipitotemporal gyrus gyrus fusiformis anterior	RFG-A (G)	42	Right Lateral occipitotemporal gyrus gyrus fusiformis anterior	RFG-A (S)
11	Left Lateral occipitotemporal gyrus gyrus fusiformis anterior	LFG-A (G)	43	Left Lateral occipitotemporal gyrus gyrus fusiformis anterior	LFG-A (S)
12	Left Insula	LINS (G)	44	Left Insula	LINS (S)
13	Right Insula	RINS (G)	45	Right Insula	RINS (S)
14	Left Occipital lobe	LOL (G)	46	Left Occipital lobe	LOL (S)
15	Right Occipital lobe	ROL (G)	47	Right Occipital lobe	ROL (S)
16	Left Gyri parahippocampalis et ambiens posterior	LPHG-P (G)	48	Left Gyri parahippocampalis et ambiens posterior	LPHG-P (S)
17	Right Gyri parahippocampalis et ambiens posterior	RPHG-P (G)	49	Right Gyri parahippocampalis et ambiens posterior	RPHG-P (S)
18	Left Lateral occipitotemporal gyrus gyrus fusiformis posterior	LFG-P (G)	50	Left Lateral occipitotemporal gyrus gyrus fusiformis posterior	LFG-P (S)
19	Right Lateral occipitotemporal gyrus gyrus fusiformis posterior	RFG-P (G)	51	Right Lateral occipitotemporal gyrus gyrus fusiformis posterior	RFG-P (S)
20	Left Medial and inferior temporal gyri posterior	LM&ITG-P (G)	52	Left Medial and inferior temporal gyri posterior	LM&ITG-P (S)
21	Right Medial and inferior temporal gyri posterior	RM&ITG-P (G)	53	Right Medial and inferior temporal gyri posterior	RM&ITG-P (S)
22	Left Superior temporal gyrus posterior	LSTG-P (G)	54	Left Superior temporal gyrus posterior	LSTG-P (S)
23	Right Superior temporal gyrus posterior	RSTG-P (G)	55	Right Superior temporal gyrus posterior	RSTG-P (S)
24	Left Cingulate gyrus anterior	LCG-A (G)	56	Left Cingulate gyrus anterior	LCG-A (S)
25	Right Cingulate gyrus anterior	RCG-A (G)	57	Right Cingulate gyrus anterior	RCG-A (S)
26	Left Cingulate gyrus posterior	LCG-P (G)	58	Left Cingulate gyrus posterior	LCG-P (S)
27	Right Cingulate gyrus posterior	RCG-P (G)	59	Right Cingulate gyrus posterior	RCG-P (S)
28	Left Frontal lobe	LFL (G)	60	Left Frontal lobe	LFL (S)
29	Right Frontal lobe	RFL (G)	61	Right Frontal lobe	RFL (S)
30	Left Parietal lobe	LPL (G)	62	Left Parietal lobe	LPL (S)
31	Right Parietal lobe	RPL (G)	63	Right Parietal lobe	RPL (S)

## Data Availability

Open dataset (dHCP)
